# Integrating transcriptomics and metabolomics to analyze the defense response of *Morus notabilis* to mulberry ring rot disease

**DOI:** 10.3389/fmicb.2024.1373827

**Published:** 2024-03-12

**Authors:** Qianqian Qian, Xinqi Deng, Sumbul Mureed, Yujie Gan, Danping Xu, Xie Wang, Habib Ali

**Affiliations:** ^1^College of Life Science, China West Normal University, Nanchong, China; ^2^College of Forestry, Sichuan Agricultural University, Chengdu, China; ^3^Institute of Agricultural Resources and Environment, Sichuan Academy of Agricultural Sciences, Chengdu, China; ^4^Department of Agricultural Engineering, Khwaja Fareed University of Engineering and Information Technology, Rahim Yar Khan, Pakistan

**Keywords:** *Morus notabilis*, mulberry ring rot disease, flavonoid biosynthesis, transcriptomics, metabolomics

## Abstract

**Introduction:**

The mulberry industry has thrived in China for millennia, offering significant ecological and economic benefits. However, the prevalence of mulberry ring rot disease poses a serious threat to the quality and yield of mulberry leaves.

**Methods:**

In this study, we employed a combination of transcriptomic and metabolomic analyses to elucidate the changes occurring at the transcriptional and metabolic levels in *Morus notabilis* in response to this disease infestation. Key metabolites identified were further validated through in vitro inhibition experiments.

**Results:**

The findings revealed significant enrichment in Kyoto Encyclopedia of Genes and Genomes pathways, particularly those related to flavonoid biosynthesis. Notably, naringenin, kaempferol, and quercetin emerged as pivotal players in *M. notabilis*’ defense mechanism against this disease pathogen. The upregulation of synthase genes, including chalcone synthase, flavanone-3-hydroxylase, and flavonol synthase, suggested their crucial roles as structural genes in this process. *In vitro* inhibition experiments demonstrated that kaempferol and quercetin exhibited broad inhibitory properties, while salicylic acid and methyl jasmonate demonstrated efficient inhibitory effects.

**Discussion:**

This study underscores the significance of the flavonoid biosynthesis pathway in *M. notabilis*’ defense response against mulberry ring rot disease, offering a theoretical foundation for disease control measures.

## Introduction

*Morus notabilis* is one of the varieties of mulberry belonging to the wild mulberry germplasm resources. Mulberry is a deciduous tree or shrub, native to China, widely distributed in tropical and temperate regions, and is an economically and ecologically versatile tree species (Yu et al., 2021; Feng et al., 2023; Xing et al., 2023). Ecologically, mulberry is a typical tufted mycorrhizal plant with a well-developed root system that reduces surface runoff, and it is widely used in ecological restoration of rocky desertification to cope with the problem of soil erosion (Wen et al., 2020; Xing et al., 2023). It is also tolerant to heavy metals in contaminated soil and can improve the activity and diversity of microorganisms in contaminated soil (Zeng et al., 2020). Economically, mulberry leaves have been used to feed silkworms and as animal fodder for livestock, and the fruits can be made into a variety of foods (Chan et al., 2016). Mulberry also has a wide range of medicinal values. Flavonoids are the main components of mulberry and have a variety of biological activities such as antioxidant, antimicrobial, antidiabetic, anti-obesity, etc. (Chan et al., 2016). The fruit of mulberry is also used as an analgesic, anthelmintic, hypotensive, hypoglycemic, laxative, etc. (Batiha et al., 2023). However, mulberry is susceptible to biotic and abiotic stresses and is infested by many pests and pathogens, leading to the development of various types of diseases in mulberry that severely limit the growth of mulberry. Mulberry may be caused by pathogenic microorganisms such as fungi, bacteria, actinomycetes, nematodes, and viruses (Liu, 2020). Nearly 200 species of mulberry diseases have been reported in China, with fungal diseases dominating, accounting for about 80% of mulberry diseases (Pu et al., 2012a). Pu et al. summarized the fungal disease species on mulberry with 53 species of Ascomycotina (Pu et al., 2012a), 74 species of Deuteromycotina (Pu et al., 2012b), 27 species of Basidiomycotina (Pu et al., 2012c), and 1 species of Mastigomycotina (Pu et al., 2012c).

*Morus notabilis* mainly distributed in the mountainous areas with an altitude of 1,300–2,800 m in Sichuan and Yunnan Provinces of China (Xuan et al., 2014). Mulberry ring rot disease of *M. notabilis* is frequent in China, Japan, and India and other subtropical and temperate regions, is currently one of the major diseases affecting sericulture production in Asia (Yin et al., 2019; Shilin et al., 2021). Mulberry ring rot disease is a fungal disease of Ascomycotina that occurs on *M. notabilis* leaves (Pu et al., 2012c). Takahashi et al. (1980) first reported this disease in Japan in 1980. Maji et al. (1999) first identified and reported this disease in India in 1999. China began to report research on this disease in the 1980s, and the first official report of a mulberry verticillium disease disaster in the Baise sericultural area of Guangxi was made in 1994 (Xie et al., 2020).

Mulberry ring rot disease infects mulberry leaves, which are the main source of food for silkworms, so the widespread spread of the disease could seriously affect sericulture development. In a previous report, Xie et al. (2020) explored the fungal community structure within and around mulberry ring rot disease spots by high-throughput sequencing analysis and found significant differences between the core area of the spot, the edge area of the spot, and the uninfected area around the spot. Shilin et al. (2021) explored the intra-canopy divergence of mulberry leaf surface bacterial community structure in mulberry ring rot disease-incident areas, and found that there were significant differences in the diversity, structural composition, and function of the upper and lower surface bacterial communities of mulberry leaves within the canopy of mulberry ring rot disease-incident areas. In this work, we explored the defense response of *M. notabilis* to the pathogen mulberry ring rot disease through a combined multi-omics analysis. The Mulberry research team at Southwest University first described the genome of *M. notabilis* in 2013 (He et al., 2013), and then reported the first gap-free reference genome of Mulberry in 2023 (Ma et al., 2023). This allowed us to study *M. notabilis* from a genetic point of view. In recent years multi-omics co-analysis methods are often used in studies on plant and pathogen infections, e.g., accumulation of total flavonoid content is induced in wheat resistance to powdery mildew (Xu et al., 2023); induction of energy metabolism and nitrogen mobilization in *Phaseolus vulgaris* L. roots infected with *Fusarium oxysporum* f. sp. *phaseoli* (Chen et al., 2019); after picking peach fruits were infested with *Monilinia fructicola* for 12 h, jasmonic acid responded to the infestation as a signaling molecule, and salicylic acid responded to the infestation as a signaling molecule 48 h after infestation (Cheng et al., 2023). Currently, research on multiple fungal diseases of mulberry is dominated by the isolation and identification of pathogenic fungi and their control (Maji et al., 2005; ; Ghosh et al., 2016; Bao et al., 2020). Therefore, the study of the molecular mechanism of mulberry fungal infection and pathogenesis using transcriptomics and metabolomics is of great significance to the development of the mulberry industry.

## Materials and methods

### Plant materials

**FIGURE 1:**
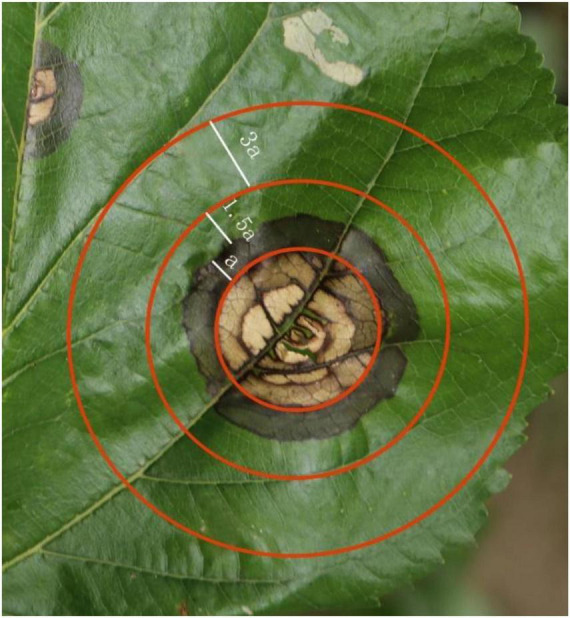
Infestation of mulberry ring rot disease-affected *Morus notabilis* gardens in in Qingshen County. HT, the core area of the lesion; LT, the marginal region of the lesion; NT, the uninfected area around the lesion; a is the width of the outermost whorl.

*Morus notabilis* leaves were obtained from mulberry ring rot disease-affected *M. notabilis* gardens in Qingshen County, Meishan City, Sichuan Province. A total of 10 diseased *M. notabilis* were randomly selected at the time of harvesting, and 10 mature leaves with mulberry ring rot disease spots were taken from each *M. notabilis*. The surface of the leaves was sterilized with 95% ethanol, then rinsed with aquae sterilization and dried with sterile filter paper. The spatial location on the leaves was divided (Figure 1) so that the area covered by all the whorls in and within the second circle from the outside to the inside was the heart zone (HT zone), which usually showed a dead yellow color. The outermost spot whorl cirri (width a) and its connected outer cirri (1.5a) were the limbic zone (LT zone), which showed both brown and green cirri. The area outside the LT zone of the lesion (3a) is the non-transmissible zone (NT), which usually shows a green color. The three zones corresponded to the pre, mid, and post periods of infection by the mulberry ring rot disease pathogen. The undeveloped, limb and core zones of each leaf were cut separately with a sterile scalpel, taking only one spot from each area. The samples were divided into three different samples according to their positions and named HT, LT, and NT. Samples were taken at the same positions of healthy leaves as control (CK). All samples were immediately frozen in liquid nitrogen and later stored in a −80°C refrigerator.

### Metabolome analysis

The sample was ground with liquid nitrogen, weighed 0.1 g, placed in a conical flask, precision added 75% methanol by volume 50 ml, weighed, ultrasonicated (power 250 W, frequency 40 kHz) for 20 min, cooled, then weighed, and made up for the loss of weight with 75% methanol by volume, and filtered through 0.22 μm microporous filtration membrane, and the filtrate was injected into the UHPLC-MS/MS system for analysis (Zhou, 2017). The sample was injected into the column with a 17 min linear gradient and a flow rate of 0.2 ml/min. The chromatographic gradient elution program is shown in Supplementary Table 1. The Orbitrap Q Exactive™ HF-X mass spectrometer was operated in positive and negative polarity mode, with the Spray Voltage set to 3.2 kV, the Sheath gas flow rate set to 40 arb, the Capillary Temp set to 320°C and the Auxiliary Gasflow rate was set to 10 arb.

### Transcriptome analysis

Each sample had 1 μg of total RNA and was used as an RNA sequencing sample preparation as input material for generating sequencing libraries. A total of 12 RNA-Seq libraries were constructed with four treatments of HT, LT, NT, and CK, with three replicates for each combination. RNA integrity and total amount were assessed using the RNA Nano 6000 Assay Kit from the Bioanalyzer 2100 System (Agilent Technologies, CA, USA). Library preparations were sequenced on the Illumina Novaseq platform and 150 bp paired-end reads were generated. Raw reads in fastq format were processed through in-house perl scripts to obtain high-quality clean reads, and all subsequent analyses were based on the clean reads. The data quality of the sample sequencing outputs was assessed by calculating Q20, Q30, and GC. The clean reads after quality control were compared to the reference genome. The mulberry reference genome and gene model annotation files were downloaded directly from the genome website.^1^ The index of the reference genome was built using Hisat2 (version 2.1.0),^2^ and the locus information of the reads was obtained by using Himat2 to compare the paired-end clean reads to the reference genome (Meng, 2019). All genes were annotated for gene function based on GO (Gene Ontology) and KEGG (Kyoto Encyclopedia of Genes and Genomes) databases.

### Expression analysis of differential genes

The FPKM of each gene was calculated based on the length of the gene and the reads mapped to that gene, and the relative expression of the transcripts was estimated based on the FPKM (Bray et al., 2016). Two-by-two comparisons were performed from the RNA-Seq data, i.e., HT vs. CK, LT vs. CK, and NT vs. CK. Differential expression analysis was performed for each sample based on the FPKM values of the genes in each group using the DESeq2 R package (version 1.20.0) with an adjusted *p*-value < 0.05 and | log2(Foldchange)| ≥ 0 as criteria for screening differential genes (DEGs), which were used for GO and KEGG pathway enrichment analysis.

### *In vitro* antimicrobial test

Since the isolation and purification of *Spondylocladium mori* (Sawada) are difficult, the test organism used in the *in vitro* inhibition test was *Corynespora cassiicola*, which was isolated and identified from mulberry whorl spots, and the test strain was provided by Sichuan Academy of Agricultural Sciences. The mycelia were inoculated into potato dextrose agar (PDA) medium for 3 days with inoculation needles and left to be used. Before the experiment was carried out, the pre-test proved that the organic solvent dimethyl sulfoxide (DMSO) would not inhibit the bacteria. The bacteriostatic substance was then configured into a 3 mg/ml solution based on molecular polarity. Autoclaved 30 ml of PDA was added to the solution and poured into petri dishes (90 mm × 20 mm). The solution was cooled and inoculated with *C. cassiicola* grown for 72 h. Colonies were measured and photographed at 24 h intervals. Three biological replicates were performed for each treatment. In this study, 11 metabolites for *in vitro* antibacterial test were screened based on the enrichment of DAMs under the pathway by combining data from transcriptome and metabolome assays (Supplementary Table 2).

### Statistical analysis

Data from principal component analysis (PCA) were transformed with metaX software and then plotted with Origin 2022. Cluster heatmaps were plotted using the R package Pheatmap and metabolite data were normalized with *z*-score. The R package ggplot2 was used to plot bubble plots.

## Results

### Quality evaluation of sequencing raw data

In this experiment, Illumina sequencing was used to transcriptionally sequence 12 *M. notabilis* leaf samples, and the quality control of raw reads was performed through raw data filtering, Phred score calculation (Q20 and Q30), sequencing error rate checking, and GC content distribution checking to obtain clean reads for use in subsequent analysis (Table 1). A total of 82.3G clean sequences were obtained, and the average clean sequences of CK, HT, LT, and NT were 7.05G, 6.91G, 6.69G, and 6.79G, respectively, with an error rate of 0.03%, an average of 97.65% for Q20, 93.37% for Q30, and an average of 46.19% for GC content, which indicated that the data were reliable and could be used for subsequent analysis. In addition, the relatively low Mapping rate of HT samples might be caused by too much variation of the samples relative to the reference genome at the late stage of infestation.

**TABLE 1:** Summary of raw RNA-Seq data quality.

Sample	Raw reads	Clean reads	Clean bases (G)	Error rate(%)	Q20 (%)	Q30 (%)	GC percent (%)	Reads mapped	Mapping rate (%)
CK1	45,979,360	45,161,798	6.77	0.03	97.83	93.73	45.82	32,629,197	72.25
CK2	49,264,706	48,765,640	7.31	0.03	97.76	93.54	46.33	35,903,595	73.62
CK3	47,686,560	47,143,072	7.07	0.03	97.74	93.53	46.31	34,140,062	72.42
HT1	47,265,840	46,561,186	6.98	0.03	97.51	93.3	46.45	29,030,495	62.35
HT2	47,632,250	47,083,002	7.06	0.03	97.79	93.67	47.74	13,996,004	29.73
HT3	45,225,012	44,629,830	6.69	0.03	97.82	93.77	48.29	21,501,824	48.18
LT1	42,925,402	42,261,414	6.34	0.03	96.85	91.64	45.74	26,686,841	63.15
LT2	45,491,388	44,924,780	6.74	0.03	97.76	93.58	45.95	30,984,141	68.97
LT3	47,119,182	46,511,502	6.98	0.03	97.7	93.54	45.17	27,817,390	59.81
NT1	45,292,242	44,689,980	6.70	0.03	97.56	93.17	45.13	30,154,911	67.48
NT2	46,003,232	45,407,118	6.81	0.03	97.72	93.47	45.62	32,543,854	71.67
NT3	46,505,998	45,678,922	6.85	0.03	97.73	93.48	45.72	32,274,818	70.66
Average	46,365,931	45,734,854	6.86		97.65	93.37	46.19		
Total	556,391,172	548,818,244	82.3						

### Screening for differentially expressed genes

**FIGURE 2:**
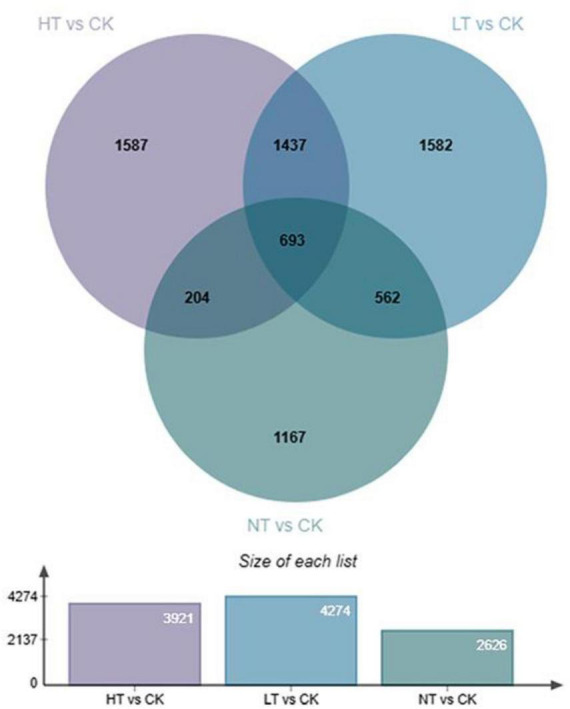
Venn diagram of the number of DEGs for the three comparison groups.

A total of 16,293 DEGs were detected in the six comparison groups. A total of 3,921 DEGs were detected in HT vs. CK (2,405 up and 1,516 down); a total of 4,274 DEGs were detected in LT vs. CK (2,547 up and 1,727 down); and a total of 2,626 DEGs were detected in NT vs. CK (1,619 up and 1,007 down) (Figure 2). This shows that significant changes occurred in the middle and late stages of infection compared with the pre-infection stage, which may be related to the increase of disease resistance pathways. The differential genes in HT vs. CK were slightly lower than those in LT vs. CK, which may be caused by the dieback condition in the center of the leaf spots of *M. notabilis* in the late stage of infection.

### GO annotation

To further understand the function of DEGs, we performed GO functional annotation of DEGs in different partitions. We presented the 30 most significant terms in bar charts according to the three categories of biological processes (BP), cellular components (CC), and molecular functions (MF), as well as the up and down of DEGs (Supplementary Figure 1). The results showed that HT vs. CK DEGs were mainly involved in biological processes such as “response to biotic stimulus,” “defense response,” “carbohydrate derivative catabolic process,” and “photosynthesis,” etc. The cellular components are mainly enriched in processes such as “photosystem,” “photosynthetic membrane,” “thylakoid,” “thylakoid part,” etc. Mainly involved in molecular functions such as “heme binding,” “tetrapyrrole binding,” “oxidoreductase activity,” “iron ion binding,” etc. The DEGs screened by LT vs. CK were mainly involved in the following biological processes: “defense response,” “response to biotic stimulus,” “response to stress,” “carbohydrate derivative catabolic process.” It is mainly involved in “cell periphery,” “cell wall,” “external encapsulating structure,” and “tethering complex.” Mainly involved in molecular functions such as “tetrapyrrole binding,” “heme binding,” “enzyme inhibitor activity,” and “oxidoreductase activity” and so on. The DEGs screened by NT vs. CK were mainly involved in biological processes such as “multi-organism process,” “cell recognition,” “pollination,” and “pollen-pistil interaction.” It is mainly involved in “DNA packaging complex,” “protein-DNA complex,” “thylakoid,” and “thylakoid part” in cellular components. In molecular functions, it is mainly involved in “transcription regulator activity,” “DNA binding transcription factor activity,” “heme binding,” “tetrapyrrole binding,” etc. This suggests that the defense response of *M. notabilis* is not strong in the early stage of the disease, but in the middle and late stage of the infection, it is a direct defense through “response to biotic stimulus,” “defense response,” and “response to stress,” and at the same time, the resistance is increased through the regulation of metabolism level in the plant.

### KEGG annotation

**FIGURE 3:**
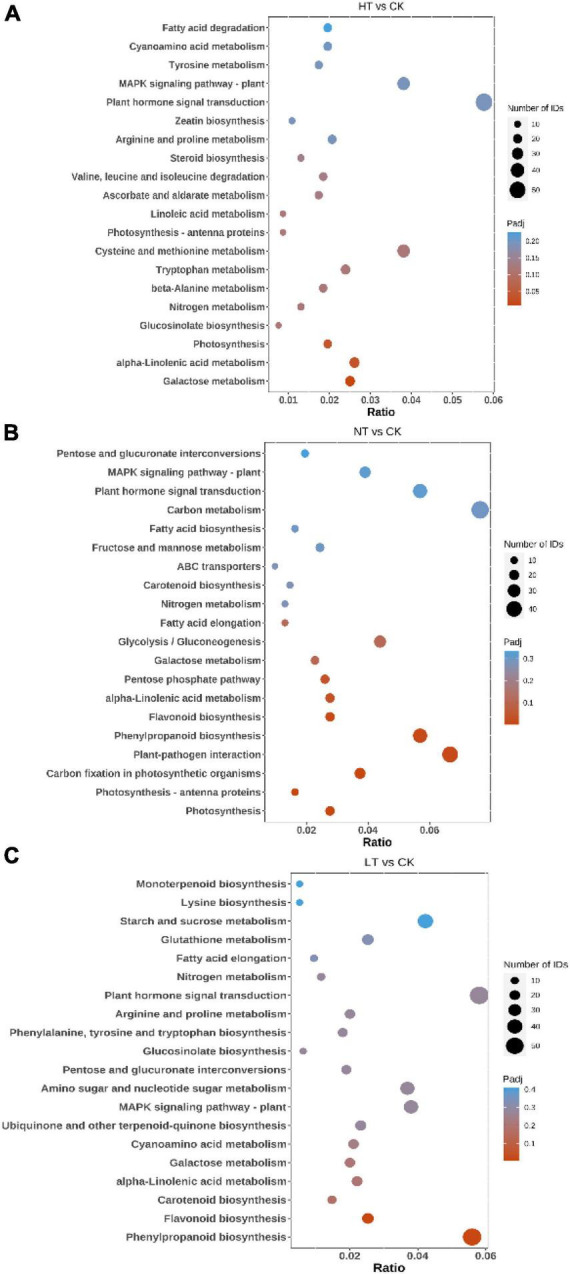
Top 20 pathways enriched in KEGG by DEGs. **(A)** HT vs. CK; **(B)** LT vs. CK; and **(C)** NT vs. CK.

Kyoto Encyclopedia of Genes and Genomes pathway analysis can further understand the biological functions and interactions of related genes (Cheng et al., 2023). The results showed (Figure 3) that HT vs. CK differential genes were significantly enriched in “Galactose metabolism,” “alpha-Linolenic acid metabolism,” “Photosynthesis,” and “Glucosinolate biosynthesis” pathways. In addition, many DEGs are listed in “MAPK signaling pathway – plant,” “Tyrosine metabolism,” “Tryptophan metabolism,” “Plant hormone signal transduction,” “Cysteine and methionine metabolism,” “Flavonoid biosynthesis” and other pathways. The LT vs. CK DEGs mainly enriched in “Phenylpropanoid biosynthesis,” “Tryptophan metabolism,” “Plant hormone signal transduction,” “Cysteine and methionine,” and “Flavonoid biosynthesis” pathways. “Phenylpropanoid biosynthesis,” “Carotenoid biosynthesis,” “MAPK signaling pathway – plant,” “alpha-Linolenic acid metabolism,” “Plant hormone signal transduction,” “Glucosinolate biosynthesis” also had a large enrichment of DEGs. NT vs. CK is mainly enriched in pathways such as “Photosynthesis,” “Photosynthesis-antenna proteins,” “Carbon fixation in photosynthetic organisms,” “Plant-pathogen interaction,” “Phenylpropanoid biosynthesis,” “Flavonoid biosynthesis,” “alpha-Linolenic acid metabolism,” and “Pentose phosphate pathway.” The enrichment of the “plant-pathogen interaction” pathway indicates that plants have begun to defend against pathogenic bacteria in the early stage of infection. In response to the infection of mulberry ring rot disease, *M. notabilis* mainly involves phenylpropanoid biosynthesis, flavonoid biosynthesis, plant hormone signal transduction, and other pathways, which may be related to the resistance to pathogenic bacteria.

### Metabolomics analysis

**FIGURE 4:**
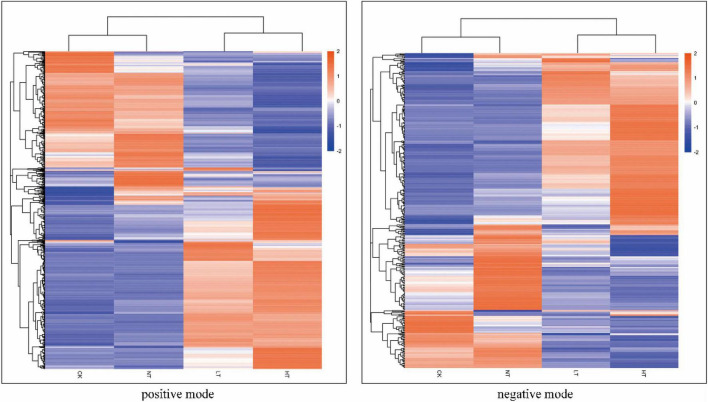
Cluster heat map of differential metabolites (positive mode on the left, negative mode on the right).

An overall clustered heat map analysis was first performed on the samples separately in the positive and negative ion modes, which revealed significant intergroup variation that could be used for the next analyses (Figure 4). PCA was then performed on each sample to determine the overall distribution trend among the different samples. As shown in Supplementary Figure 2, the contribution of the first principal component (PC1) in the positive ion mode was 59.03%, 58.05%, and 32.23% for the three regions, and the contribution of PC1 in the negative ion mode was 59.24%, 56.49%, and 39.50%, respectively; and the contribution of the second principal component (PC2) in the positive ion mode was 14.96% for the three regions, respectively, 15.86% and 29.37%, respectively, and the contribution of PC2 in the negative ion mode was 14.58%, 17.05%, and 24.87%, respectively; at the same time, all samples were significantly separated. The relationship between metabolite expression and sample species was modeled using partial least squares regression Supplementary Figure 3. The PLS-DA correlation models R2Y (cum) and Q2Y (cum) ranged from 0.99 to 1.00 and 0.59 to 0.9, respectively, among the different comparison groups under the positive and negative ion model. R2Y was larger than Q2Y, which was a better construction of the model.

**FIGURE 5:**
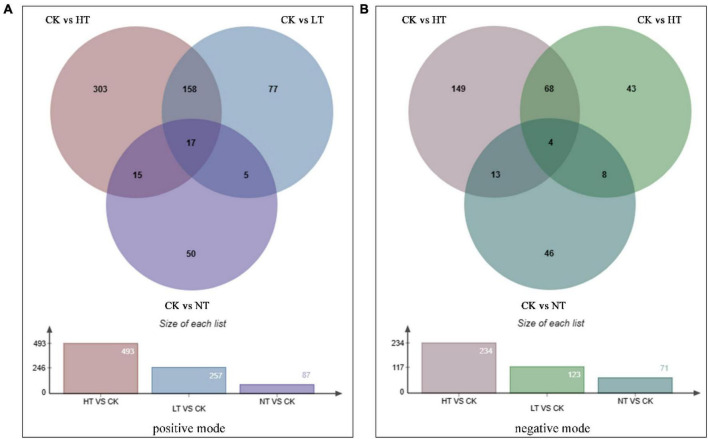
Venn diagram of differential metabolites. **(A)** Positive mode and **(B)** negative mode.

Screening for differential metabolites yielded a total of 625 DAMs in positive ion mode (Figure 5A), with 493 (187 up, 306 down), 257 (84 up, 173 down), and 87 (25 up, 62 down) obtained from the CK vs. HT, CK vs. LT, and CK vs. NT comparator groups, respectively. The negative ion mode screened 331 DAMs, and the three comparison groups of CK vs. HT, CK vs. LT, and CK vs. NT screened 234 (66 up, 168 down), 123 (37 up, 86 down), and 71 (13 up, 58 down) species, respectively (Figure 5B). As can be seen from the Wayne diagram, there were 17 differential metabolites common to the three regions in the positive ion mode, 303 species accumulated only in the HT region, 77 species accumulated only in the LT region, and 50 species accumulated only in the NT region. There were four differential metabolites common to the three regions in the negative ion mode, 149 species accumulated only in the HT region, 43 species accumulated only in the LT region, and 46 species accumulated only in the NT region. In both positive and negative ion modes, DAMs were most abundant in CK vs. HT, followed by CK vs. LT, which was consistent with the transcriptome results. It suggests that *M. notabilis* has the strongest defense response against pathogens in the middle and late stages of infection.

### Differential metabolite KEGG pathway enrichment analysis

**FIGURE 6:**
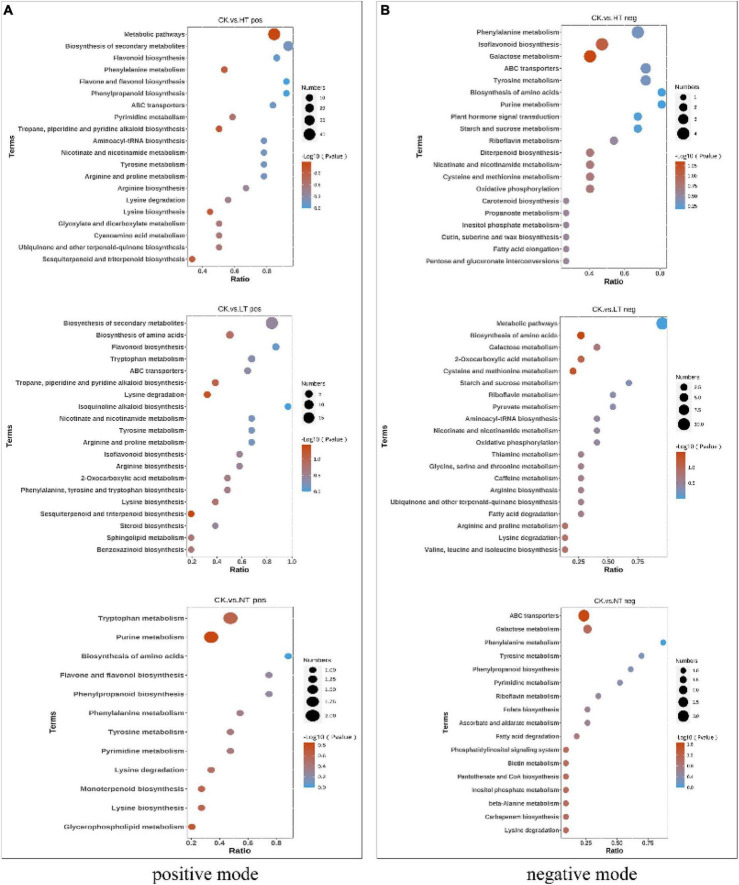
Kyoto Encyclopedia of Genes and Genomes enrichment bubble diagram. **(A)** Positive mode and **(B)** negative mode.

Kyoto Encyclopedia of Genes and Genomes enrichment analysis of DAMs (Figure 6) showed that HT vs. CK pos enriched a total of 43 metabolite pathways, including “Metabolic pathways,” “Biosynthesis of secondary metabolites,” “Flavonoid biosynthesis,” “Phenylalanine metabolism,” “Flavone and flavonol biosynthesis,” “Phenylpropanoid biosynthesis” and other pathways were more abundant. LT vs. CK pos enriched a total of 33 metabolite pathways, “Biosynthesis of secondary metabolites,” “Biosynthesis of amino acids,” “Flavonoid biosynthesis,” “Tryptophan metabolism,” “ABC transporters,” “Tropane, piperidine, and pyridine alkaloid biosynthesis” were enriched in large quantities. NT vs. CK pos had the least enrichment pathway, and a total of 12 metabolite pathways were enriched, including “Tryptophan metabolism,” “Biosynthesis of amino acids,” “Phenylpropanoid biosynthesis,” “Flavone and flavonol biosynthesis,” “Purine metabolism,” and other pathways. In negative ion mode HT vs. CK neg, LT vs. CK neg, and NT vs. CK neg were enriched in 35, 24, and 17 pathways, respectively. The flavonoid biosynthesis pathway was significantly enriched in both the mid- and late stages of infection, which is consistent with the results of transcriptomics analysis.

### Analysis of flavonoid biosynthetic pathways

**FIGURE 7:**
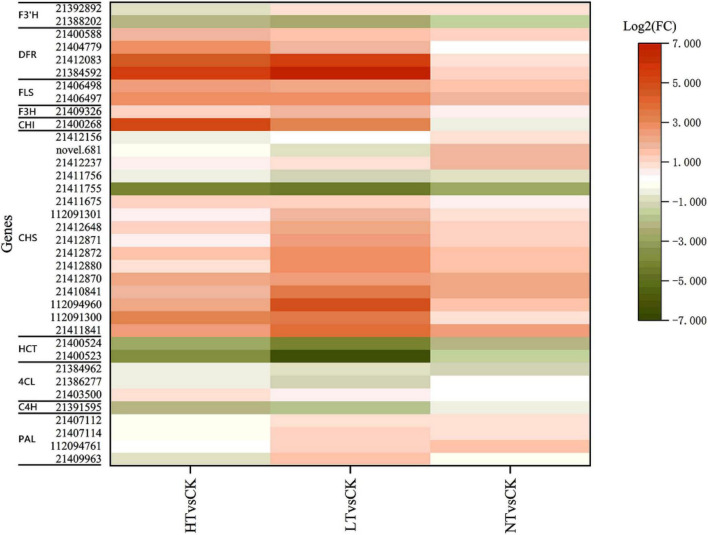
Profiles of differentially expressed genes in flavonoid biosynthesis pathways. F3′H, flavonoid 3′-hydroxylase; DFR, dihydroflavonol 4-reductase; FLS, flavonol synthase; F3H, flavanone-3-hydroxylase; CHI, chalcone isomerase; CHS, chalcone synthase; HCT, hydroxycinnamoyl transferase; 4CL, 4-coumarate:CoA ligase; C4H, cinnamic acid-4-hydroxylase; PAL, phenylalanine ammonia-lyase.

Transcriptome KEGG enrichment revealed that 36 DEGs were injected into the flavonoid biosynthesis pathway (Figure 7), including 4 PAL genes, 3 4CL genes, 1 C4H gene, 16 chalcone synthase (CHS) genes, 1 CHI gene, 1 flavanone-3-hydroxylase (F3H) gene, 4 DFR genes, 2 flavonol synthase (FLS) genes, 2 HCT genes, and 2 F3′H genes. The expression of most genes was increased compared to CK, especially CHS, F3H, and FLS, and they increased more in HT and LT. HCT and C4H genes, however, showed down. Among the DAMs, flavonoids included quercetin, kaempferol, eriodictyol, naringenin, hesperetin, apigenin-8-C-glucoside, myricetin, and their corresponding glycosides.

**FIGURE 8:**
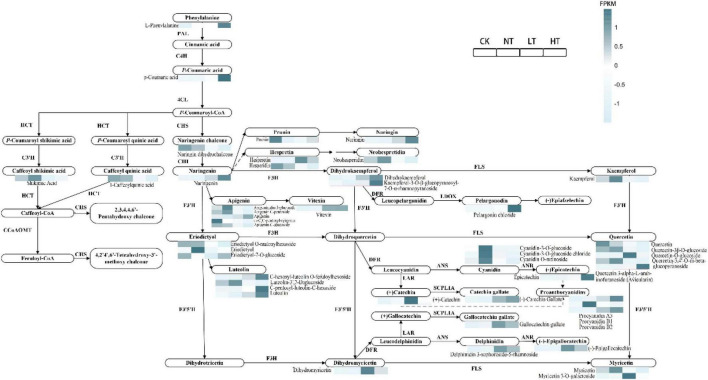
Expression networks of DAMs in the flavonoid biosynthesis pathway.

The DEGs and DAMs associated with the flavonoid biosynthesis pathway were mapped to the KEGG pathway (Figure 8). Based on the expression of metabolites it can be seen that most of the metabolites showed upregulation compared to CK. L-phenylalanine, p-coumaric acid, naringenin, hesperetin, dihydrokaempferol, kaempferol-3-O-β-glucopyranosyl-7-O-α-rhamnopyranoside, apigenin, vitexin, pelargonin chloride, quercetin-O-glucoside, quercetin 3-alpha-L-arabinofuranoside (Avicularin), C-hexosyl-luteolin O-feruloylhexoside, C-pentosyl-luteolin-C-hexoside, luteolin, (+)-catechin, (−)-catechin gallate, gallocatechin gallate, procyanidin A3, procyanidin B1, procyanidin B2, dihydromyricetin, delphinidin 3-sophoroside-5-rhamnoside, (−)-epigallocatechin, myricetin, myricetin 3-O-galactoside, and epicatechin, metabolites that showed up in LT or HT. Shikimic acid, neohesperidin, kaempferol, apigeninidin 5-glucoside, apigenin C-pentoside, eriodictyol, cyanidin-3-O-glucoside, cyanidin-3-O-glucoside chloride, cyanidin O-rutinoside, and quercetin showed up in NT compared to CK. Only naringin dihydrochalcone, 1-caffeoylquinic acid, prunin, apigenin C-glucoside, hesperidin, naringin, di-C, C-pentosyl-apigenin, eriodictyol O-malonylhexoside, and eriodictyol-7-O-glucoside, all of these metabolites showed down compared to CK. Most of the metabolites were significantly upregulated in the middle and late stages of infection, and some of them were also significantly upregulated in the early stages of infection, suggesting that flavonoid metabolites play a role in the defense against mulberry ring rot disease.

### *In vitro* antimicrobial test of metabolites

**FIGURE 9:**
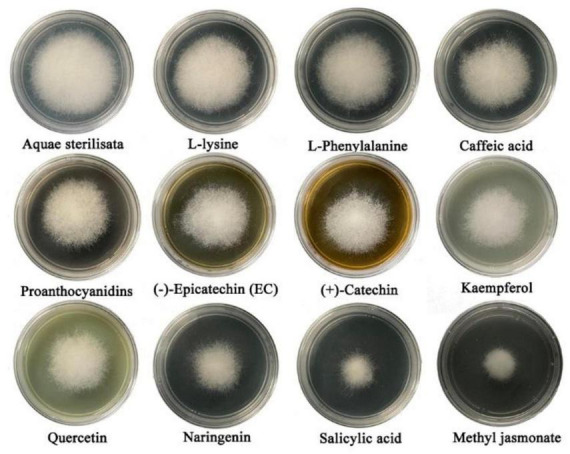
*In vitro* antimicrobial test of metabolites on *Corynespora cassiicola* after 48 h.

**FIGURE 10:**
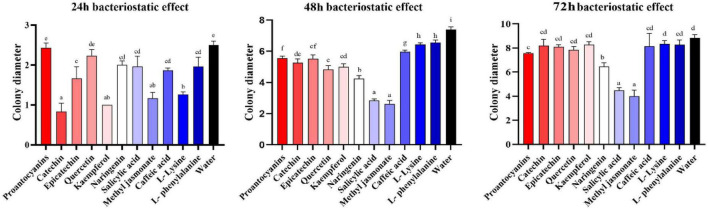
Colony diameter at 24, 48, and 72 h after inoculation. The result is the mean of the three biological replicates ±SE, *n* = 3. Lower case letters represent significant differences, *p* < 0.05.

Metabolomics analysis revealed significant accumulation of *M. notabilis* flavonoid compounds after infection by the mulberry ring rot disease pathogen, therefore we selected seven metabolites related to flavonoid biosynthesis pathway, L-phenylalanine, proanthocyanidins, (−)-epicatechin (EC), (+)-catechin, quercetin, naringenin, and kaempferol for *in vitro* antimicrobial test in *C. cassiicola*. In addition literature suggests that phenylpropanoid biosynthesis and plant hormone signal transduction also play an important role in plant disease resistance (Bao et al., 2020), also in the present study the enrichment of both pathways was found, therefore L-lysine and caffeic acid associated with phenylpropanoid biosynthesis, and salicylic acid and methyl jasmonate associated with plant hormone signal transduction were selected to be used for the *in vitro* antibacterial test along with the phenylpropanoid biosynthesis. After 48 h of inoculation, all nine metabolites showed strong inhibitory effects on *C. cassiicola*, except for two amino acids, L-phenylalanine, and L-lysine, which had low antimicrobial properties (Figure 9). Significance analysis of colony diameter at 24, 48, and 72 h showed (Figure 10) that kaempferol and (+)-catechin had the best inhibitory effect at 24 h of inoculation, and proanthocyanidins had the worst inhibitory effect. L-lysine and caffeic acid showed strong inhibitory effect at 24 h, and weaker inhibitory effect at invasive naringenin and salicylic acid showed the opposite inhibitory effect, which was significantly greater than most of the other substances at 48 and 72 h, and the inhibitory effect also showed a trend of gradual enhancement; methyl jasmonate showed strong inhibitory effect throughout the whole process; moreover, the inhibitory effect of 24 and 48 h (−)-epicatechin (EC) was the best. The (−)-epicatechin (EC) and (+)-catechin inhibited *C. cassiicola* better than proanthocyanidins at 24 and 48 h, and proanthocyanidins produced higher inhibition than (−)-epicatechin (EC) and (+)-catechin at 72 h.

## Discussion

Flavonoid synthesis plays a key regulatory role in plant growth and development and has been demonstrated in plant-fungus interactions (Jiang et al., 2016). It was shown that the red side of red mango (*Mangifera indica*) inhibited *Colletotrichum gloeosporioides* better than the green side because of the higher accumulation of anthocyanosides and flavonols, etc., on the red side (Sudheeran et al., 2020). The analysis of key transcripts and metabolites of *Camellia oleifera* cultivars with different resistance showed that these DEGs were significantly enriched in tyrosine metabolism, phenylpropanoid synthesis and flavonoid synthesis, and metabolomics studies were carried out, and the results showed that flavonoid synthesis has great potential in controlling anthracnose (Yang et al., 2022). In this work, KEGG results of transcriptomics and metabolomics of infected mulberry leaves revealed enrichment of flavonoid biosynthesis pathway compared to CK, so we analyzed the expression levels of genes and metabolites related to this pathway. It was found that F3H, FLS, and DFR genes were significantly up after infestation of mulberry, and that quercetin, kaempferol, and proanthocyanidins all accumulated after mulberry leaves were infected with the disease, which is in agreement with the results of previous studies. It has been reported that upregulation of flavonoid biosynthesis genes using genetic modifications can enhance the accumulation of flavonoid compounds and consequently resistance, for example, upregulation of F3H, FLS, DFR, ANS, and ANR genes elevated the level of quercetin, kaempferol, and proanthocyanidin accumulation and improved the defense of *Populus alba* against *Dothiorella gregaria* infection (Bai et al., 2020). Therefore, the metabolism of flavonoids, which is likely to be an important component of plant defense responses, is also central to the expression of plant disease resistance genes (Xinqi et al., 2022).

Although some secondary metabolites are not directly involved in plant growth and development, they can still serve as chemical defenses, such as lipids (Lim et al., 2017), flavonoids (Cushnie and Lamb, 2011), alkaloids (Yang et al., 2017), polyphenols (Hosseinzadeh et al., 2016), and glycosides (Escobedo-Martínez et al., 2010). That is, they can be used for defense against damage caused by plant diseases under specific environmental and physiological conditions. Flavonoids are widely found in plants as substances with defense functions. For example, in maize DIMBOA (2,4-dihydroxy-7-methoxy-2H-1,4-benzoxazin-3(4H)-one) is resistant to fungi (Frey et al., 1997). Lignin resistance to *F. oxysporum* f. sp. *medicaginis* is closely related to the isoflavone pathway (Gill et al., 2017). Isoflavones also have antimicrobial functions (Undhad et al., 2020).

The phenylpropanoid biosynthetic pathway involves the biogenesis of a variety of phenolic polymers, such as flavonoids and phenolic acids, which play important roles as structural and signaling molecules in plant development and defense (Yadav et al., 2020). In the present study DAMs were found to be enriched in flavonoid and phenylpropanoid biosynthetic pathways, which is consistent with previous reports that significant accumulation of flavonoids and phenolic compounds is associated with plant-pathogen disease resistance, e.g., table grapes showed enhanced disease resistance to gray mold due to increased accumulation of phenolic and flavonoids (Xu et al., 2019). Similarly, phenylpropanoid metabolism enhanced resistance of tobacco to tobacco mosaic virus (Shadle et al., 2003), Cherry Tomato fruit to black mold (Wei et al., 2017) and cotton to *Verticillium* wilt via (Li et al., 2019).

In this study, the major flavonoid DAMs included quercetin, kaempferol, eriodictyol, naringenin, hesperidin, vitexin, myricetin, and their corresponding glycosides. Vitexin has been reported to regulate the hydrophobicity of the surface of *Staphylococcus aureus*, thereby inhibiting its growth (Das et al., 2022). Kaempferol is found in *Chelidonium majus* aqueous, which is a natural antifungal product (Balkan et al., 2017). Methyl jasmonate, salicylic acid, naringenin, and quercetin were also found to have strong inhibitory effects on *C. cassiicola* in the antibacterial test. In a previous study, methyl jasmonate, an important phytohormone, was found to inhibit the expansion of the diameter of kiwifruit (*Actinidia chinensis*) *Botryosphaeria dothidea* lesion (Li et al., 2022). Salicylic acid has long been proved to be a natural antimicrobial compound (Barman et al., 2014). Naringenin was able to inhibit the formation of the second and third phases of *Streptococcus mutans* biofilm second and third stage formation, thus achieving the effect of bacterial inhibition (Yue et al., 2018). Quercetin concentration of 0.25 mg/ml, *Penicillium expansum* mycelial structure was severely damaged, and it has a positive effect on kiwifruit post-harvest blue mold control (Zhang et al., 2018). Taken together, the accuracy of the results of the antibacterial test can also be confirmed. Therefore, the metabolites enriched in *M. notabilis* samples may contribute to the resistance of *M. notabilis* to mulberry ring rot disease, and the enhancement of *M. notabilis* disease resistance through the interaction of these metabolites with the pathogen provides a direction for studies analyzing the function of metabolites.

## Conclusion

In this study, metabolomic and transcriptomic analyses unveiled the activation of the flavonoid biosynthetic pathway in *M. notabilis* infested with mulberry ring rot disease. The transcriptional expression of structural genes, including PAL, C4H, 4CL, HCT, CHS, CHI, F3H, FLS, and DFR, was induced, leading to the accumulation of metabolites such as naringenin chalcone, naringenin, kaempferol, quercetin, and its derivatives in the metabolic flux. Notably, naringenin, kaempferol, and quercetin, emerged as key metabolites in the defense response of *M. notabilis* against mulberry ring rot disease. Simultaneously, CHS, F3H, and FLS were identified as crucial structural genes orchestrating the resistance of *M. notabilis* to mulberry ring rot disease. In addition, *in vitro* bacteriostatic tests showed that all nine compounds, except L-phenylalanine and L-lysine, had effective bacteriostatic effects. These different compounds showed the strongest inhibitory effect when infested with *C. cassiicola* for 24 h. This comprehensive investigation highlights the pivotal role of flavonoid compounds in *M. notabilis*’ defense mechanism against mulberry ring rot disease, which provides theoretical guidance for further research on *M. notabilis* resistance and breeding for disease resistance.

## Data availability statement

The original contributions presented in this study are included in this article/Supplementary material, further inquiries can be directed to the corresponding authors.

## Author contributions

QQ: Conceptualization, Formal analysis, Investigation, Methodology, Writing – original draft. XD: Methodology, Software, Writing – original draft. SM: Methodology, Writing – review & editing. YG: Methodology, Writing – review & editing. DX: Conceptualization, Funding acquisition, Investigation, Methodology, Resources, Supervision, Writing – review & editing. XW: Data curation, Funding acquisition, Investigation, Methodology, Resources, Supervision, Writing – review & editing. HA: Formal analysis, Software, Writing – review & editing.

## Footnotes

1
https://www.ncbi.nlm.nih.gov/assembly/GCF_000414095.1

2
http://www.di.fc.ul.pt/∼afalcao/hisat2_windows.html
