# Supplementary material for: Integrating transcriptomics and metabolomics to analyze the defense response of Morus notabilis to mulberry ring rot disease
Source: Front Microbiol. 2024 Mar 12;15:1373827. doi: 10.3389/fmicb.2024.1373827 (PMC10963518; doi:10.3389/fmicb.2024.1373827)
Supplement: Supplementary file 1 [file Data_Sheet_1.docx]

**Supplimentry Figures**.


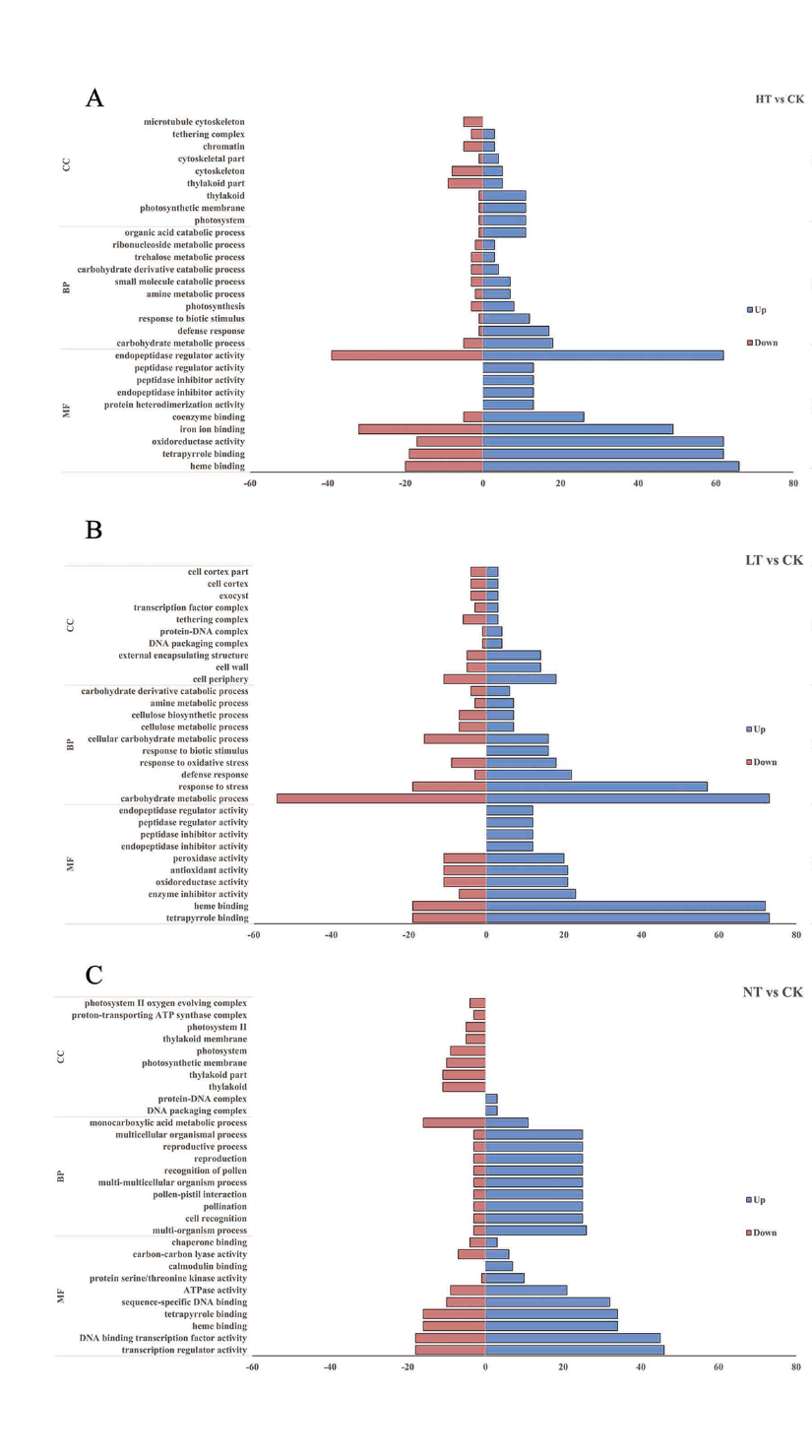


**Figure S1**. Top 30 entries enriched by GO analysis. (A) HT vs CK; (B) LT vs CK; (C) NT vs CK.


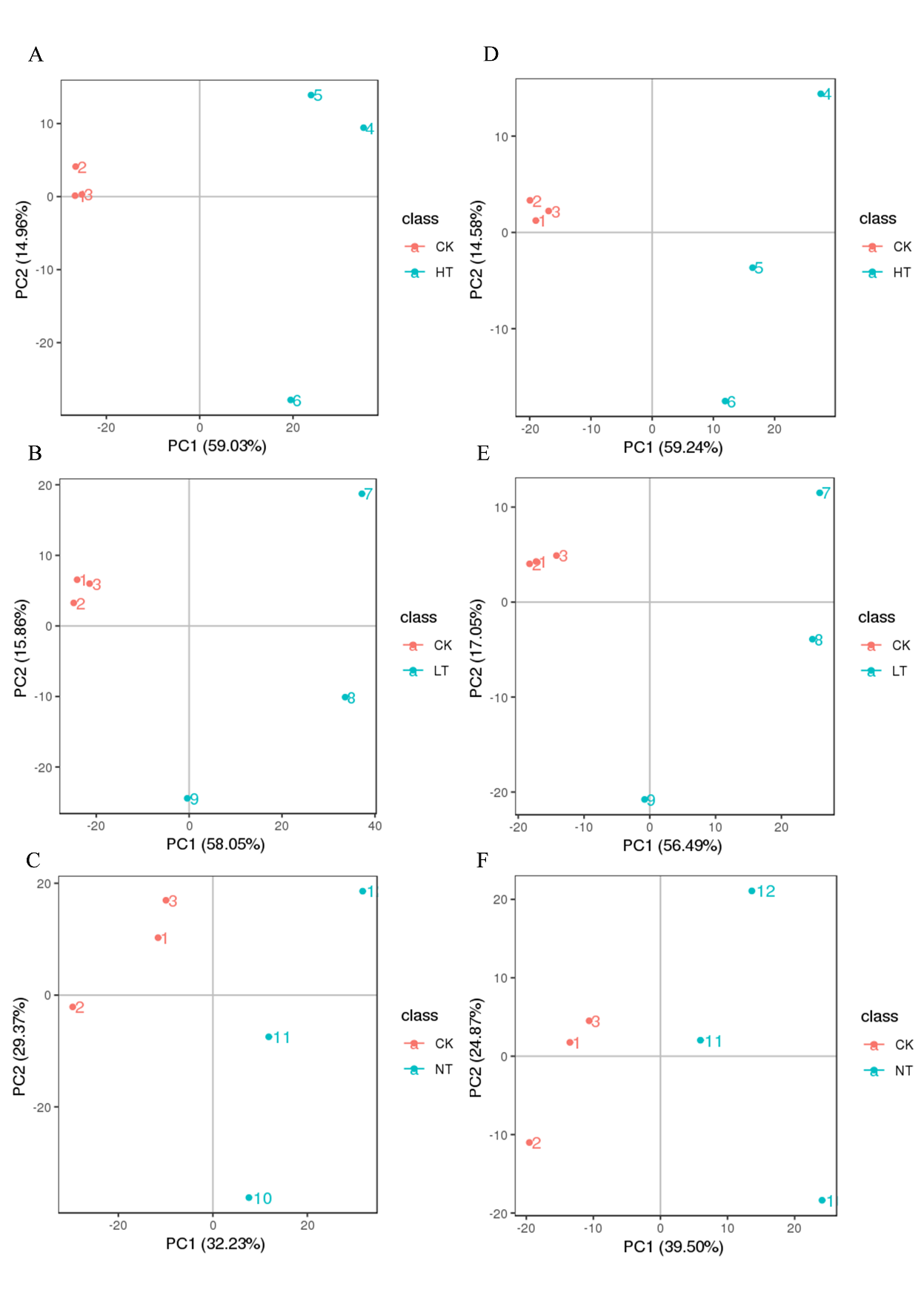


**Figure S2**. Metabolite PCA analysis. A, B, and C are positive ion patterns, and D, E, and F are negative ion patterns. (A) and (D) CK vs HT; (B) and (E) CK vs LT; (C) and (F) CK vs NT.


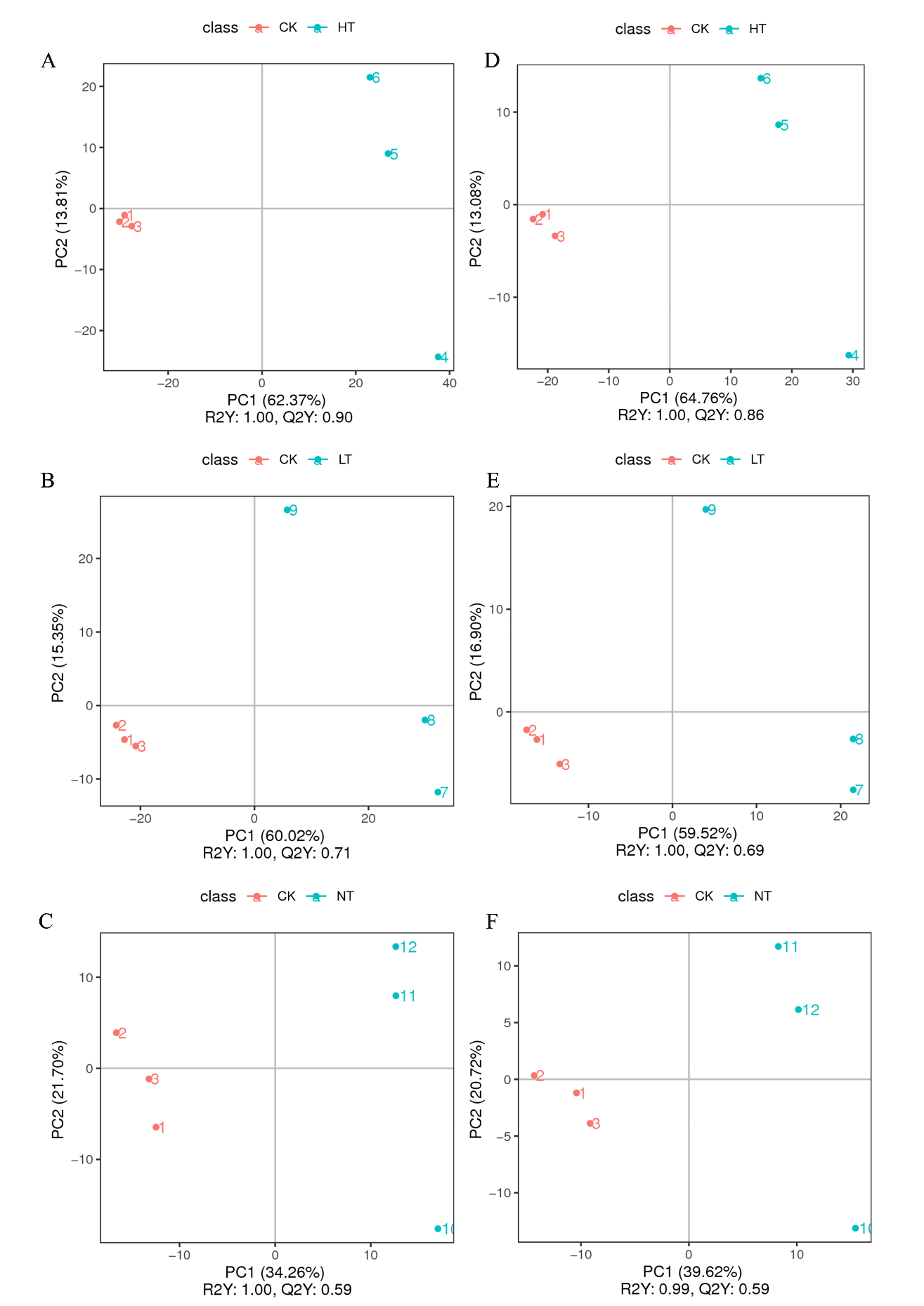


**Figure S3**. Metabolite PL-SDA analysis. A, B, and C are positive ion patterns, and D, E, and F are negative ion patterns. (A) and (D) CK vs HT; (B) and (E) CK vs LT; (C) and (F) CK vs NT.
