# Supplementary material for: Integrating transcriptomics and metabolomics to analyze the defense response of Morus notabilis to mulberry ring rot disease
Source: Front Microbiol. 2024 Mar 12;15:1373827. doi: 10.3389/fmicb.2024.1373827 (PMC10963518; doi:10.3389/fmicb.2024.1373827)
Supplement: Supplementary file 2 [file Table_1.docx]

**Supplementary Tables**

Table S1 Chromatographic gradient elution program

| Time | A% | B% |
| --- | --- | --- |
| 0 | 98 | 2 |
| 1.5 | 98 | 2 |
| 12 | 0 | 100 |
| 14 | 0 | 100 |
| 14.1 | 98 | 2 |
| 17.0 | 98 | 2 |

The positive mode eluents were eluent A (0.1% formic acid in water) and eluent B (methanol). Negative mode eluents were eluent A (5 m ammonium acetate, pH 9.0) and eluent B (methanol).

**Table S2** In vitro antimicrobial test substance for *Corynespora cassiicola*

| Compounds | Primary classification of compounds | Secondary classification of compounds | CAS |
| --- | --- | --- | --- |
| L-Phenylalanine | Amino acids and derivatives | Amino acids and derivatives | 63-91-2 |
| L-lysine | Amino acids and derivatives | Amino acids and derivatives | 56-87-1 |
| Methyl jasmonate | Fatty acid derivatives | Fatty acid derivatives | 39924-52-2 |
| Salicylic acid | Phenolic acid | Phenolic acid | 69-72-7 |
| Caffeic acid | Phenolic acid | Phenolic acid | 331-39-5 |
| Naringenin | Flavonoid | Dihydroflavone | 480-41-1 |
| Kaempferol | Flavonoid | Flavonol | 520-18-3 |
| Quercetin | Flavonoid | Flavonol | 117-39-5 |
| Proanthocyanidins | Polyphenol | Flavonoid polymers | 4852-22-6 |
| (-)-Epicatechin (EC) | Flavonoid | Flavanol | 490-46-0 |
| (+)-Catechin | Flavonoid | Flavanol | 154-23-4 |
